# Epitope Mapping and MHC Restriction of Next-Generation Tuberculosis Vaccine Antigens in Mice

**DOI:** 10.3390/vaccines14100866

**Published:** 2026-10-01

**Authors:** Samuel J. Vidal, Alejandro A. Espinosa Perez, Ganad D. Neka, Aleisha M. G. Roberts, Yu Sun, Lili Benitez Angeles, Hannah I. Angulo Espinoza, Ellis A. Strutton, Esther R. Mbiwan, Jayeshbhai Chaudhari, Woojae Choi, Michelle Lifton, Pablo Penaloza-MacMaster, Dan H. Barouch

**Affiliations:** Center for Virology and Vaccine Research, Beth Israel Deaconess Medical Center, Harvard Medical School, Boston, MA 02215, USAaespinos@bidmc.harvard.edu (A.A.E.P.); ysun11@bidmc.harvard.edu (Y.S.); a00835761@tec.mx (L.B.A.); a01737054@tec.mx (H.I.A.E.); jchaudha@bidmc.harvard.edu (J.C.);

**Keywords:** tuberculosis, vaccine, cellular immunity, CD4 T cells, CD8 T cells, T cell epitopes, MHC class I, MHC class II, tetramer

## Abstract

**Background/Objectives**: Classical major histocompatibility complex (MHC)-restricted T cells play essential roles in tuberculosis (TB) immunity. Characterizing the antigen targets, dominant epitopes, and MHC restriction of protective T-cell responses is important for understanding TB immunity and next-generation TB vaccine development. **Methods**: We recently used vaccine screening in mice to identify Rv1387 (PPE20), Rv0287 (EsxG), Rv1788 (PE18), and Rv1886c (Ag85B) as protective antigens, and in this paper, we characterized the T-cell responses to these antigens in mice. **Results**: We first identified the full repertoire of CD4 and CD8 T-cell epitopes in C57BL/6 and BALB/c mice. We further defined the MHC class I and II restriction of immunodominant epitopes, enabling the development and validation of tetramers for probing cognate T-cell responses. Finally, we used these tetramers to contrast lung mucosal T-cell phenotypes in the setting of Bacillus Calmette–Guérin (BCG) and mRNA vaccination. **Conclusions**: These results define the T-cell epitopes and MHC restriction of next-generation TB vaccine antigens and contribute reagents for studying TB T-cell immunity.

## 1. Introduction

A large body of evidence supports an essential role for classical major histocompatibility complex (MHC)-restricted T cells in tuberculosis (TB) immunity, among other immune subsets [1,2]. In humans, depletion of CD4 T cells during HIV infection correlates with TB prevalence and severity [3]. Mutations that cause Mendelian susceptibility to mycobacterial disease (MSMD) are broadly associated with the T-cell IFN-γ response [4]. Disruption of T-cell TNF signaling through inborn errors of immunity or immunosuppression with TNF blockers increases mycobacterial disease burden [5]. Classical studies in mice showed that depletion of CD4 and CD8 T cells accelerates TB pathogenesis [6], and these were later corroborated by antibody depletion studies in non-human primates [7].

Rational antigen selection from a large repertoire of candidates is a major challenge in TB vaccine development [8,9]. The *Mycobacterium tuberculosis* (Mtb) genome contains approximately 4000 open reading frames (ORFs), of which over 100 have been reported to be recognized by T cells during natural infection in humans [10,11]. Accordingly, over the past three decades a variety of methods have been deployed for the identification and selection of protective antigens for subunit vaccines against TB. ESAT-6, Ag85B, and MTB32A vaccine antigens were identified as immunogenic proteins present in secreted culture filtrates [12,13]. Other strategies identified antigens expressed during chronic infection in mice [14], antigens recognized by CD8 T cells in humans [15], and antigens not shared with BCG [16], among others.

In recent years, systematic screening studies have suggested that antigens may contribute differentially to immunity. One protection screen in mice of antigens belonging to defined functional and structural groups showed a spectrum of efficacy with a subset of antigens showing enhanced protection and many showing no or minimal protection [17]. T-cell receptor (TCR)-based screening methods suggest some TCRs are enriched among Mtb-exposed individuals who do not progress to TB [18] or among clonotypes that efficiently recognize infected macrophages [19]. Immunopeptidomics of infected macrophages showed that a limited subset of T-cell epitopes are presented by MHC [20,21]. Conversely, mechanistic studies in mice show that some immunodominant T-cell antigens may function as decoys that skew T cells to non-protective responses [22]. Overall, the identification and characterization of protective antigens and their T-cell responses is important for understanding TB immunity as well as developing protective vaccines.

We recently performed a screen in mice to assess the protective efficacy of the human latent TB (LTB) CD4 antigen repertoire, and we identified Rv1387/PPE20, Rv0287/EsxG, Rv1788/PE18, and Rv1886c/Ag85B as the antigens conferring the most protection in our model [23]. In this study, we present the epitope mapping and MHC restriction of the full immunodominant T-cell repertoire of these antigens. These data facilitated the development of new tetramers that we use to contrast the mucosal T-cell response of Bacillus Calmette–Guérin (BCG) and mRNA vaccines.

## 2. Methods

### 2.1. Mouse Studies

Eight-week-old female C57BL/6 (strain #000664), BALB/c (strain #000651), and CB6F1/J (strain #100007) mice were obtained from Jackson Laboratory and housed in pathogen-free conditions at Beth Israel Deaconess Medical Center. In all experiments, dots represent individual mice. For cellular and humoral immune responses, we estimated that, to detect a 0.5 log_10_ change in vaccine-induced immune responses with 80% power and 0.05 alpha error, n = 5 mice would be needed per group. Each experimental group contained n = 4–5 mice. A total of 29 mice were used for epitope-mapping studies, 30 mice for MHC-restriction studies, n = 80 mice for tetramer-validation studies, and n = 24 mice for the mucosal immunology studies, for a total of n = 163 animals. Mice received in crates of n = 20 from the vendor were randomly assigned to vaccine groups. No exclusion criteria were implemented. Immunization and tissue harvesting were performed according to a prespecified fixed order, and antigen groups were processed sequentially in the same order. Animal age, sex, strain, source, and housing conditions were standardized across groups. Each mouse was considered a biological replicate.

### 2.2. DNA Vaccine Production

H37Rv reference sequences were obtained from Mycobrowser [24], codon-optimized for expression in *Homo sapiens*, and cloned into mammalian pcDNA3.1(+) expression plasmids (GeneArt, Thermo Fisher Scientific, Waltham, MA, USA). A subset of ORFs with a valine (V) start codon was replaced with methionine (M) for mammalian expression, and no other modifications were made to the reference primary sequence.

### 2.3. mRNA Vaccine Production

We created a custom mRNA expression vector containing optimized non-coding backbone elements spanning from a T7 promoter compatible with CleanCap^®^ AG-3′OMe (TriLink BioTechnologies, San Diego, CA, USA) through the 5′ and 3′ untranslated regions (UTRs) and a 110-nt poly(A) tail interrupted by a linker (A30LA70). Reference coding sequences [24] for Rv1387, Rv0287, and Rv1788 were cloned into plasmid DNA templates. Plasmids were linearized using restriction enzymes, purified, and subjected to T7 RNA polymerase-mediated in vitro transcription (IVT) to generate mRNA. N1-methylpseudouridine-5′-triphosphate (m1Ψ-UTP) was used to replace UTP to generate modified nucleoside-containing mRNAs, and co-transcriptional capping was performed in a one-pot reaction using CleanCap^®^ AG-3′OMe reagent. Following IVT, template DNA was removed by DNase I digestion, and mRNA was purified by LiCl precipitation and resuspended in RNase-free buffer. Microfluidic mixing with a NanoAssemblr Ignite+ system (Cytiva Lifesciences, Marlborough, MA, USA) was used to encapsulate purified RNA into lipid nanoparticles (LNPs). An aqueous RNA solution at pH 4.0 was mixed with an ethanolic lipid solution at a 3:1 aqueous:ethanol ratio. We used a lipid mixture consisting of ALC-0315, DOPE, cholesterol, and C14-PEG2000. Particle size and polydispersity were measured by dynamic light scattering (DLS) using a Zetasizer Nano (Malvern Instruments, Malvern, UK), with particle diameters typically in the range of 70–100 nm. RNA encapsulation efficiency was quantified using the Quant-iT™ RiboGreen RNA Assay Kit (Thermo Fisher Scientific, Waltham, MA, USA), which confirmed >95% encapsulation efficiency for all RNA-LNP formulations.

### 2.4. BCG Vaccine Strain

BCG Pasteur strain was a gift from the Urdahl laboratory (University of Washington, Seattle, WA, USA). BCG Pasteur was used in all BCG immunizations. For preparation of BCG vaccine stock, vaccine strain was grown in media consisting of Middlebrook 7H9 (BD DIFCO^TM^, Franklin Lakes, NJ, USA) containing 10% Middlebrook OADC (BD BBL, Sparks, MD, USA), 0.5% glycerol (Sigma Aldrich, Burlington, MA, USA), 0.05% tween 80 (Sigma Aldrich, Burlington, MA, USA), and 0.05% tyloxapol (Sigma Aldrich, Burlington, MA, USA). We pelleted cells twice with resuspension in PBS containing 0.05% tyloxapol and then pelleted a third time with resuspension in PBS containing 0.05% tyloxapol and 15% glycerol. We then passaged cells through a 40 μm filter followed by a 20 μm filter for clump removal, followed by storage at −80 °C and titering by agar outgrowth assay.

### 2.5. Mouse Immunizations

DNA vaccine immunizations were performed by diluting 50 μg of vaccine in 100 μL of phosphate-buffered saline (PBS), followed by intramuscular (IM) injection of 50 μL into each quadriceps muscle. Single antigen mRNA-LNP immunizations were performed by diluting 5 μg of mRNA in 100 μL of PBS, followed by IM injection of 50 μL into each quadriceps muscle. For BCG immunizations, a total of 1 × 10^6^ CFU of BCG Pasteur vaccine strain was diluted in 200 μL of PBS, followed by subcutaneous (SC) injection of 100 μL into each of the bilateral lower flanks.

### 2.6. Spleen and Lung Harvesting

Following euthanasia, we harvested spleens and transferred them into R10 medium containing RPMI supplemented with 10% FBS (Gibco, Grand Island, NY, USA) and 1% penicillin–streptomycin (Fisher Scientific, Waltham, MA, USA). We mechanically dissociated tissues through a 100 μm filter, and we lysed red blood cells with ACK Lysing Buffer (Gibco, Grand Island, NY, USA). We then filtered plenocytes through a 30 μm filter, pelleted, and resuspended in R10. For lung harvest, mice were euthanized and tissues were transferred into GentleMACS C tubes (Miltenyi, Gaithersburg, MD, USA) containing 4 mL of R10. This was supplemented with a 5X digestion buffer consisting of R10 supplemented with 5 mg of collagenase type I (Worthington Biochemicals, Lakewood, NJ, USA) and 0.5 mg of DNase I (Sigma, Aldrich, Burlington, MA, USA) in 1 mL of solution. Lung tissue was then mechanically dissociated using a GentleMACS instrument according to the manufacturer instructions (Miltenyi, Gaithersburg, MD, USA), followed by passaging through a 70 μm filter. The solution then underwent red blood cell lysis with ACK Lysing Buffer (Gibco, Grand Island, NY, USA), and lung cells were then filtered through a 30 μm filter. Following lymphocyte isolation, viable cell counts were quantified by trypan blue exclusion using a Countess 3 instrument (Thermo Fisher Scientific, Waltham, MA, USA).

### 2.7. Intracellular Cytokine Staining (ICS)

After single-cell preparations were made or blocking antibodies were added, we stimulated murine splenocytes with peptide (GenScript, Piscataway, NJ, USA) at 400 ng per test or DMSO as negative control for 1 h in R10 medium followed by GolgiStop/GolgiPlug (BD Biosciences, San Diego, CA, USA) overlay for 6 h at 37 °C with 5% CO_2_. Cells were then washed and stained with antibodies. Splenocytes were stained with live/dead (Aqua or NIR) and cell surface markers in MACS solution (Miltenyi, Gaithersburg, MD, USA) supplemented with 2% BSA (Miltenyi, Gaithersburg, MD, USA) and 1.5% penicillin–streptomycin (Fisher Scientific, Waltham, MA, USA) prior to permeabilization with Cytofix/Cytoperm (BD Biosciences, San Diego, CA, USA) and staining with intracellular markers in Perm/Wash (BD Biosciences, San Diego, CA, USA). We then fixed splenocytes in 2% formaldehyde and stored them at 4 °C until flow cytometry on an LSR II flow cytometer (BD Biosciences, San Diego, CA, USA). For ICS, splenocyte cell surface markers included CD3 (clone 17A2, BD Biosciences, San Diego, CA, USA), CD19 (clone 6D5, BioLegend, San Diego, CA, USA), CD4 (clone RM4-5, BioLegend, San Diego, CA, USA), CD8a (clone 53-6.7, BD Biosciences, San Diego, CA, USA), CD44 (clone IM7, BD Biosciences, San Diego, CA, USA), and CD62L (clone MEL-14, BioLegend, San Diego, CA, USA). Splenocyte intracellular markers included IFN-γ (clone XMG1.2, BioLegend, San Diego, CA, USA), and TNF (clone MP6-XT22, BioLegend, San Diego, CA, USA). We quantified antigen-specific T-cell responses as the percentage of CD44^hi^ cells producing IFN-γ, TNF, or IL-2 within the respective CD4^+^ or CD8^+^ T-cell populations.

### 2.8. MHC Class I and Class II Blocking Studies

After single-cell preparations were made, murine splenocytes were incubated with blocking antibodies against MHC class I or class II (BioLegend, San Diego, CA, USA; Invitrogen, Waltham, MA, USA; or Vector Labs, Newark, CA, USA) at a concentration of 10 μg/mL or with the corresponding isotype control. Antibodies were diluted in PBS and added to the R10 medium at a 1:3 ratio before incubation for 1 h at 37 °C with 5% CO_2_, with resuspension after 30 min. Cells were subsequently stimulated with the corresponding peptide as below. Blocking antibodies included I-A^d^ (clone 34-5-3S, Invitrogen, Waltham, MA, USA), I-E^d^ (clone 34-1-4S, Vector Labs, Newark, CA, USA), I-A/I-E (clone M5/114.15.2, BioLegend, San Diego, CA, USA), IgG2a isotype control (clone MOPC-173, Invitrogen, Waltham, MA, USA), IgG2b, κ isotype control (clone RTK4530, BioLegend, San Diego, CA, USA), and IgM isotype control (clone 11E10, Vector Labs, Newark, CA, USA). Antigen-specific T-cell responses were quantified as the percentage of CD44^hi^ cells producing IFN-γ or IFN-γ and TNF within the respective CD4^+^ or CD8^+^ T-cell populations.

### 2.9. MHC Class I and II Tetramer and Cell Surface Antibody Staining

MHC class I H-2K^d^:PPE20_155-164_ (NIH Tetramer Core Facility, Atlanta, GA, USA) or MHC class II I-A^b^:PPE20_1-16_, I-A^b^:EsxG_46-61_, I-A^b^:PE18_1-16_ (NIH Tetramer Core Facility) or class II control I-A^b^:CLIP_87-101_ (NIH Tetramer Core Facility, Atlanta, GA, USA) were used to detect Mtb-specific murine CD8 and CD4 T cells in lung and spleen. MHC class I and class II tetramers were provided by the NIH Tetramer Core Facility (Emory University, Atlanta, GA, USA). Single-cell suspensions were stained with a single tetramer at concentrations recommended by the NIH Tetramer Core Facility guidelines for CD4 tetramers and incubated at 37 °C for 2 h, with resuspension every 30 min. After tetramer staining, surface staining was conducted at 4 °C for 30 min. Cells were then fixed in 2% formaldehyde and stored at 4 °C until flow cytometry on an LSRII flow cytometer (BD Biosciences, San Diego, CA, USA).

Antigen-specific CD8 and CD4 T cells were identified by gating on live/dead, dump channel negative cells (CD19^+^ and CD11c^+^), CD8^+^ or CD4^+^ T cells, and tetramer^+^ cells. For tetramer staining experiments, cell surface markers included CD8a (clone 53-6.7, BD Biosciences, San Diego, CA, USA), CD127 (clone SB/199, BD Biosciences, San Diego, CA, USA), CX3CR1 (clone Z8-50, BD Biosciences, San Diego, CA, USA), KLRG1 (clone 2F1, BioLegend, San Diego, CA, USA), CD19/CD11c dump channel (clones 6D5/N418, BioLegend, San Diego, CA, USA), CD44 (clone IM7, BioLegend, San Diego, CA, USA), CD62L (clone MEL-14, BioLegend, San Diego, CA, USA), CD4 (clone RM4-5, BioLegend), CXCR3 (clone CXCR3-173, BD Biosciences, San Diego, CA, USA), CD103 (clone M290, BD Biosciences, San Diego, CA, USA), PD-1 (clone 29F.1A12, BioLegend, San Diego, CA, USA), and CD69 (clone H1.2F3, BD Biosciences, San Diego, CA, USA). Finally, mice were infused with 5 μg of CD45 antibody (clone 30-F11, BioLegend, San Diego, CA, USA) diluted in a total volume of 200 μL PBS by tail vein injection and euthanized 5–10 min later. Tetramer-positive antigen-specific T-cell responses were quantified as the percentage of CD44^hi^ tetramer (APC)^+^ cells within the respective CD4^+^ or CD8^+^ T-cell populations.

### 2.10. Quantification and Statistical Analysis

Flow cytometry data were analyzed using FlowJo 10.10.0 software. Statistical comparisons between groups for immunogenicity studies were performed with GraphPad Prism 9.4.0 software. Most studies contained five biological replicates to provide 80% power to detect a minimum 2-fold difference with less than 0.05 alpha error.

## 3. Results

### 3.1. CD4 and CD8 T-Cell Epitope Mapping

We previously identified the immunodominant T-cell epitopes of Rv1387, Rv0287, Rv1788, and Rv1886c in CB6F1 mice, a first filial (F1) cross of the parent C57BL/6 and BALB/c strains [23]. We first mapped the epitopes to the parent C57BL/6 and BALB/c strains using DNA vaccines with splenocyte ex vivo overlapping peptide pool stimulation followed by flow cytometry and ICS, as performed previously [25,26]. We used the same 16mer overlapping by 11 peptide libraries from our previous studies [23] (Table S1).

Given the large size of Rv1387 (539 amino acids) and evidence of multiple T-cell epitopes in the CB6F1 strain [23], we used a matrix subpooling strategy to reduce the number of peptide stimulations required while maintaining single-peptide resolution. The peptide library was organized into two sets of overlapping pools (A–K and L–U), such that each peptide was represented in two unique pools (Table S2). Candidate epitopes were identified by intersecting positive responses between the two pool sets and subsequently confirmed using individual peptides in a second deconvolution experiment. In C57BL/6, CD4 subpools A, H, and L-O were positive (Figure S1A), and deconvolution confirmed CD4 epitopes at peptides 1 and 72/73 (Figure 1A). There were no Rv1387 CD8 epitopes in C57BL/6 (Figure S1B). In BALB/c, CD4 subpools A, C, H, O, T, and U were positive (Figure S1C), and deconvolution confirmed immunodominant CD4 epitopes at peptides 9/10, 28/29, and 74 (Figure 1B). BALB/c CD8 subpools D, L, and M were positive (Figure S1D), and deconvolution confirmed an immunodominant CD8 epitope at peptide 31 (Figure 1C).

Given the small size of Rv0287 (97 amino acids) and Rv1788 (99 amino acids), these antigens were mapped in single experiments. For Rv0287, C57BL/6 showed an immunodominant CD4 epitope at peptide 10 (Figure 1D), and BALB/c showed an immunodominant CD4 epitope at peptide 12 as well as other subdominant CD4 epitopes (Figure 1E). These Rv0287 CD4 epitopes in C57BL/6 and BALB/c matched the immunodominant CD4 region in CB6F1 at peptides 10–12 [23]. For Rv1788, C57BL/6 showed an immunodominant CD4 epitope at peptide 1 (Figure 1F), whereas BALB/c showed subdominant CD4 epitopes at peptides 1 and 13 (Figure 1G). We observed no CD8 epitopes in either parent strain for Rv0287 and Rv1788, consistent with prior data in CB6F1 [23].

Finally, given the intermediate size of Rv1886c (325 amino acids), we used a linear subpooling strategy from our previous studies [23] (Table S1). In C57BL/6, CD4 subpools E, H, L, and M were positive (Figure S1E), and deconvolution confirmed CD4 epitopes at peptides 24, 37, 57, and 61 (Figure 1H). Similar to RV1387, there were no Rv1886c CD8 epitopes in C57BL/6 (Figure S1F). In BALB/c, CD4 subpools A, F, and M were positive (Figure S1G), and deconvolution confirmed immunodominant CD4 epitopes at peptides 1, 29/30, and 61/62 (Figure 1I). BALB/c CD8 subpools E and H were strongly positive (Figure S1H), and deconvolution confirmed immunodominant CD8 epitopes at peptides 21/22 and 37 (Figure 1J). Thus, overall, we mapped the full repertoire of immunodominant T-cell epitopes of Rv1387, Rv0287, Rv1788, and Rv1886c in the C57BL/6 and BALB/c strains.

### 3.2. MHC Class I and II Restriction

Our epitope mapping studies revealed an Rv1387 CD8 epitope that is restricted to the BALB/c MHC I alleles (Figure 1C). Given that this Rv1387 response is not well-characterized, we first defined the minimal CD8 epitope. We used a walking 9mer approach spanning Rv1387 peptide 31. Interestingly, the NetMHCpan-4.1 machine learning algorithm [27] predicted a primary 10mer peptide (TYSTVSRSAL) restricted to H-2K^d^ (Table S3). Vaccination of BALB/c mice with Rv1387 DNA vaccine and splenocyte stimulation with the walking 9mers and 10mer confirmed the 10mer as the primary minimal CD8 epitope (Figure 2A). In addition, MHC I-specific blocking antibody studies confirmed that the 10mer was restricted to H-2K^d^ (Figure 2B).

We additionally observed Rv1886c CD8 epitopes in BALB/c mice centered at epitopes 21/22 and 37 (Figure 1J). Notably, three 9mers were previously reported in those regions [28,29]: YYQSGLSIV (peptide 21), MPVGGQSSF (peptide 22), and IYAGSLSAL (peptide 37). However, the MHC restriction of these 9mers remains incompletely understood. NetMHCpan-4.1 predicted restriction to H-2K^d^ (YYQSGLSIV and IYAGSLSAL) and H-2L^d^ (MPVGGQSSF, Table S4). We performed MHC class I-specific blocking antibody studies with these 9mers and confirmed the predicted restriction (Figure 2C–E).

We previously reported that the immunodominant Rv0287 CD4 epitope in CB6F1 mice is at peptide 12 [23], and in this paper, we found that this peptide is restricted to the BALB/c MHC II alleles (Figure 1E), which include I-A^d^ and I-E^d^. Interestingly, NetMHCpan-4.1 predicted two distinct CD4 epitopes within peptide 12 binding to I-A^d^ and I-E^d^ (Table S5). Indeed, individual I-A^d^ and I-E^d^ antibody blocking antibodies failed to inhibit T-cell activation by peptide 12, whereas a combined I-A/E^d^ blocking antibody abrogated activation (Figure 2F). Together with the machine learning prediction (Table S4), these data support the presence of two overlapping BALB/c CD4 epitopes at this site.

### 3.3. Rv1387 and Rv1788 Tetramer Validation

We recently showed that a trivalent vaccine candidate comprised of Rv1387, Rv0287, and Rv1788 augmented and exceeded BCG protection against Mtb challenge in murine aerosol challenge models [23]. Tetramer reagents enable the isolation of live antigen-specific T cells for functional assays as well as improved quantification and phenotyping of these subsets. An Rv0287 C57BL/6 peptide 10 CD4 tetramer was previously reported [30]; therefore, we next characterized new tetramers for Rv1387 and Rv1788.

We previously reported that the immunodominant Rv1387 CD4 epitope in CB6F1 mice is at peptide 1 [23]. Here we show that this CD4 epitope maps to the C57BL/6 parent strain (Figure 1A). The C57BL/6 strain is null at the MHC class II I-E locus, and all CD4 epitopes in C57BL/6 are restricted to the MHC class II I-A^b^ allele. We synthesized a tetramer using the Rv1387/PPE20 peptide 1 16mer with the MHC class II I-A^b^ allele (I-A^b^:PPE20_1-16_). We first confirmed by splenocyte ICS that C57BL/6 mice vaccinated with Rv1387 produce a CD4 response to peptide 1 (Figure 3A). Moreover, these mice showed robust I-A^b^:PPE20_1-16_ staining (Figure 3B), whereas both naïve and BALB/c mice vaccinated with Rv1387 showed no background staining (Figure 3C,D).

Rv1387 stimulated a robust CD8 response in BALB/c mice, which we mapped to a peptide 31 10mer that is restricted to the MHC class I H-2K^d^ allele (Figure 1C). We therefore synthesized a tetramer using the Rv1387/PPE20 peptide 31 10mer with the MHC I H-2K^d^ allele (H-2K^d^:PPE20_155-164_). We first confirmed by splenocyte ICS that BALB/c mice vaccinated with Rv1387 produced a CD8 response to the peptide 31 10mer (Figure 3C). BALB/c mice vaccinated with Rv1387 showed robust H-2K^d^:PPE20_155-164_ staining (Figure 3D), whereas both naïve and C57BL/6 mice vaccinated with Rv1387 showed no background staining (Figure 3A,B).

We previously reported that the immunodominant Rv1788 CD4 epitope in CB6F1 mice is at peptide 1 [23]. In this paper, we show that this CD4 epitope maps to the C57BL/6 parent strain (Figure 1F). Given that the C57BL/6 strain is null at the MHC II I-E locus as above, we synthesized a tetramer using the Rv1788/PE18 peptide 1 16mer with the MHC class II I-Ab allele (I-A^b^:PE18_1-16_). We first confirmed by splenocyte ICS that C57BL/6 mice vaccinated with Rv1788 produced a CD4 response to peptide 1 (Figure 3E). C57BL/6 mice vaccinated with Rv1788 showed robust I-A^b^:PE18_1-16_ staining (Figure 3F). Naïve C57BL/6 splenocytes showed modest background (Figure 3F), whereas BALB/c mice vaccinated with Rv1387 showed no background (Figure 3G,H).

We previously reported an immunodominant Rv0287 CD4 epitope region in CB6F1 mice at peptides 10–12 [23]. In this paper, we show that this epitope region maps to peptide 10 in C57BL/6 and peptide 12 in BALB/c (Figure 1). The Rv0287 CD4 epitope at peptide 10 in C57BL/6 and a corresponding 15mer tetramer (I-A^b^:EsxG_46-61_) were previously reported [31]. We confirmed by splenocyte ICS that C57BL/6 vaccinated with Rv0287 produced a CD4 response to Rv0287 peptide 12 and that I-A^b^:EsxG_46-61_ staining is restricted to C57BL/6 and not BALB/c (Figure S2).

Finally, TB subunit vaccine studies are commonly performed in the CB6F1 strain that leverages increased MHC diversity [14,23]. We therefore examined whether the trivalent vaccine antigen tetramers to Rv1387, Rv0287, and Rv1788 demonstrate the expected staining patterns in CB6F1. Indeed, vaccinated CB6F1 mice showed the expected ICS stimulation and tetramer staining after vaccination with Rv1387 (I-A^b^:PPE20_1-16_ and H-2K^d^:PPE20_155-164_, Figure 4A,B), Rv0287 (I-A^b^:EsxG_46-61_, Figure 4C,D), and Rv1788 (I-A^b^:PE18_1-16_, Figure 4E,F). Overall, these data report and validate MHC class I and class II tetramers to Rv1387 and an MHC class II tetramer to Rv1788. They further validate tetramer staining for Rv1387, Rv0287, and Rv1788 in CB6F1 mice.

### 3.4. Mucosal Immunology of BCG^mem^ and mRNA Vaccine-Elicited T Cells

We next investigated whether our epitope mapping, MHC restriction, and tetramer validation data could be used for mucosal immunology studies in mice immunized with clinical vaccine platforms. We investigated two vaccination strategies (Figure 5A). First, we vaccinated CB6F1 mice with monovalent mRNA vaccines expressing Rv1387, Rv0287, and Rv1788, as previously described [23], and harvested lungs 2 weeks post-boost. We transitioned from the DNA to the mRNA vaccine platform because both were protective in our previous work [23] and the mRNA platform is more clinically relevant. Second, we adopted a previously described BCG memory (BCG^mem^) model [32] in which mice are vaccinated with BCG Pasteur and lungs were harvested after 52 weeks to model better the typically prolonged delay between childhood BCG vaccination and burden of TB in adolescents and adults. The BCG^mem^ model leveraged our recent observation that BCG elicits an immunodominant response to Rv1788 in the lungs of CB6F1 mice [23].

We observed robust tetramer staining in the lungs of all CB6F1 mice following both monovalent mRNA-LNP and BCG^mem^ vaccination, and there was no background in the lungs of naïve mice (Figure 5A–C and Figure S3). In addition, our tetramer protocol included pre-necropsy intravenous administration of fluorophore-conjugated CD45 as well as a flow cytometry panel with additional markers, enabling localization of T cells within the vasculature vs. lung parenchyma and additional phenotyping. We observed that Rv1788-specific T cells in BCG^mem^ mice demonstrated increased levels of differentiation (KLRG1, Figure 5E) and vascular sequestration (CX3CR1, Figure 5F) that were previously associated with poor homing to the lung parenchyma [31]. Indeed, Rv1788-specific T cells in BCG^mem^ mice showed decreased lung tissue localization compared to Rv1788—as well as Rv1387- and Rv0287-specific T cells elicited by mRNA-LNP vaccination (Figure 5G). There were insufficient lung lymphocytes in these mice for concurrent cytokine staining.

## 4. Discussion

Classical MHC-restricted T-cell responses are essential for TB immunity [1,2]. Therefore, characterizing the antigen targets, immunodominant epitopes, and MHC restriction of protective T-cell responses is important for both understanding TB immunity and developing next-generation TB vaccines. In this paper, we defined the immunodominant T-cell epitope repertoire and MHC restriction of four candidate TB vaccine antigens, Rv1387 (PPE20), Rv0287 (EsxG), Rv1788 (PE18), and Rv1886c (Ag85B), in C57BL/6 and BALB/c mice. These studies facilitated the development of tetramer reagents that we used to contrast the antigen-specific mucosal vaccine immunology of BCG and mRNA. These data advance our understanding of protective vaccine antigens and contribute reagents for studying TB T-cell immunity in mice.

The immunodominant CD4 T-cell epitopes previously observed in CB6F1 mice [23] were found to be roughly evenly distributed between the parent C57BL/6 and BALB/c strains. We confirmed that Rv1387 and Rv1886c each contain five or more regions of CD4 recognition. In contrast, the CD8 T-cell epitopes previously observed in CB6F1 mice for the Rv1387 and Rv1886c antigens were found to be solely restricted to the parent BALB/c MHC class I alleles, particularly H-2K^d^. These results suggest that many CD4 and CD8 T-cell responses may not be present in the C57BL/6 strain and support the use of more MHC-diverse strains for antigen studies. The development of monoclonal antibodies to these next-generation antigens would help confirm the in vivo levels and kinetics of these antigens with various vaccine platforms.

We found evidence that some CD4 T-cell epitope regions show promiscuous binding to multiple MHC alleles in mice, similar to MHC class II binding to CD4 TB antigens in humans [33]. For example, Rv1788 peptide 1 was shown to be positive in both C57BL/6 and BALB/c. In addition, the region of CD4 recognition spanning peptides 10–12 in Rv0287 was found to be surprisingly epitope-dense with up to three epitopes restricted to all three MHC II alleles present in the CB6F1 strain, and Rv0287 peptide 12 was found to potentially bind both I-A^d^ and I-E^d^ alleles.

Previous studies have deployed multiple methodologies to characterize some of the T-cell epitopes to these four antigens in humans. These methodologies have included genome-scale computational epitope prediction [34], TCR sequencing [18,19], and immunoprecipitation of MHC class II molecule–peptide complexes [20,21]. Interestingly, Rv0287 peptide 10 and Rv1788 peptide 1 were previously reported as regions of CD4 T-cell recognition in these studies [19,20,34], potentially reflecting orthologous MHC class II molecules and binding motifs [35,36]. Further epitope mapping in humans and other mouse strains may reveal additional regions of overlapping CD4 recognition, although cross-species differences in antigen processing as well as MHC and TCR genetics likely limit the scope of epitope conservation. Moreover, recent elegant studies dissecting recognition of autologous infected macrophages by CD4 T cells in vitro found that T-cell activation may differ by TCR: epitope combination within the same antigen [19], suggesting that TCR:epitope identity within antigens may be an important determinant of protection. It is currently unknown to what extent currently available data from either mouse vaccine protection models or human in vitro CD4 T-cell activation models predict protection in the clinical context. Overall, an approach to antigen down-selection incorporating both model systems may be optimal.

We recently showed that BCG vaccination stimulates an immunodominant CD4 T-cell response to Rv1788 in the mouse lung [23]. We produced and validated tetramers to Rv1387 (I-A^b^:PPE20_1-16_ and H-2K^d^:PPE20_155-164_) and Rv1788 (I-A^b^:PE18_1-16_), validated a previously reported CD4 tetramer to Rv0287 (I-A^b^:EsxG_46-61_), and used these tetramers to characterize antigen-specific lung mucosal T-cell responses to these antigens delivered with the mRNA vaccine platform and contrasted them to Rv1788-specific responses elicited by BCG. The BCG response showed markers of differentiation (KLRG1^+^) and vascular sequestration (CX3CR1^+^) together with poor lung parenchymal homing (IV-CD45^−^). Interestingly, KLRG1^+^/CX3CR1^+^ CD4 T cells were previously shown during natural infection in mice to exhibit poor lung parenchymal homing and protection in adoptive transfer studies [31]. These mucosal immunology features may explain how mRNA vaccines enhance BCG immunity in mice [23]. Finally, tetramer development may facilitate the isolation of antigen-specific T cells, generation of TCR-transgenic mice, and dissection of T cell-dependent mechanisms of immunity [37,38].

## 5. Conclusions

Classical MHC-restricted T cells are a primary mechanism of immunity against TB, and the characterization of protective antigen-specific responses may improve our under-standing of immunity as well as help guide vaccine development. We characterized the immunodominant epitopes and MHC restriction of four candidate TB vaccine antigens and validated new tetramer reagents for these antigens in mice. These data and reagents contribute to the understanding of protective immunity in preclinical models of TB, and future studies may provide context for these findings in human immunity against TB.

## Figures and Tables

**Figure 1:**
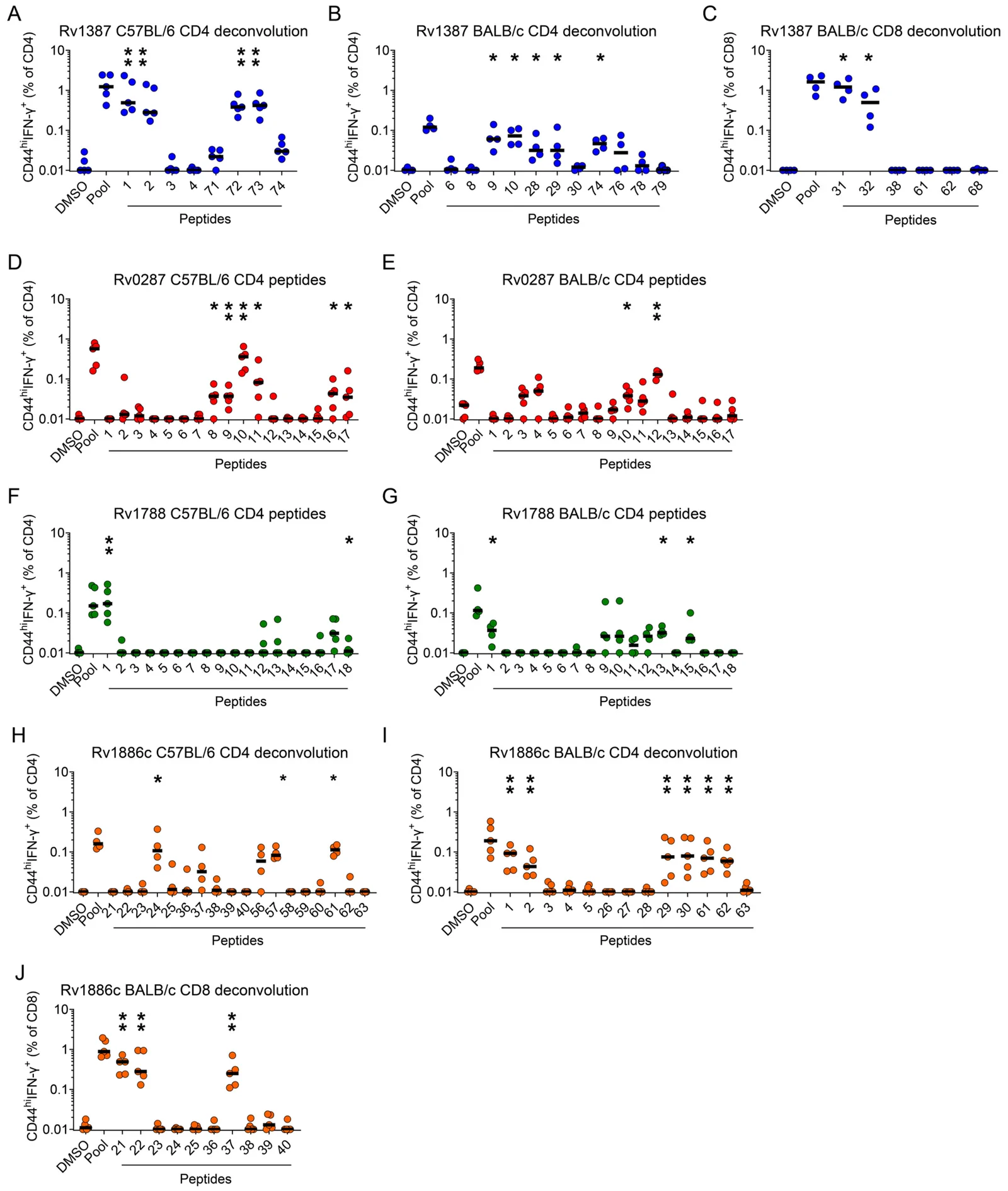
Immunodominant CD4 and CD8 T-cell epitope repertoire of the protective antigens Rv1387, Rv0287, Rv1788, and Rv1886c in C57BL/6 and BALB/c mice. (**A**) Rv1387 matrix subpool deconvolution of individual peptides inducing CD4 T-cell responses in C57BL/6 mice after 50 μg Rv1387 DNA prime-boost vaccination, ex vivo splenocyte stimulation, and flow cytometry with ICS. (**B**) Rv1387 matrix subpool deconvolution of individual peptides CD4 T-cell responses in BALB/c mice. (**C**) Rv1387 matrix subpool deconvolution of individual peptides inducing CD8 T-cell responses in BALB/c. Single peptide reactivity analysis of CD4 T-cell responses to Rv0287 in C57BL/6 (**D**) and BALB/c (**E**) mice. Single-peptide reactivity analysis of CD4 T-cell responses to Rv1788 in C57BL/6 (**F**) and BALB/c (**G**) mice. (**H**) Rv1886c CD4 linear subpool deconvolution of individual peptides inducing CD4 T-cell responses in C57BL/6 mice. (**I**) Rv1886c CD4 linear subpool deconvolution of individual peptides inducing CD4 T-cell responses in BALB/c mice. (**J**) Rv1886c CD4 linear subpool deconvolution of individual peptides inducing CD8 T-cell responses in BALB/c mice. For all experiments, groups of n = 4–5 mouse splenocytes stimulated with the indicated peptides were compared to DMSO negative control and overlapping peptide pool positive control. Dots represent individual mice and bars indicate medians. *p* values represent Mann–Whitney U tests for the indicated peptide compared to DMSO control. * Represents *p* < 0.05 and ** represents *p* < 0.01.

**Figure 2:**
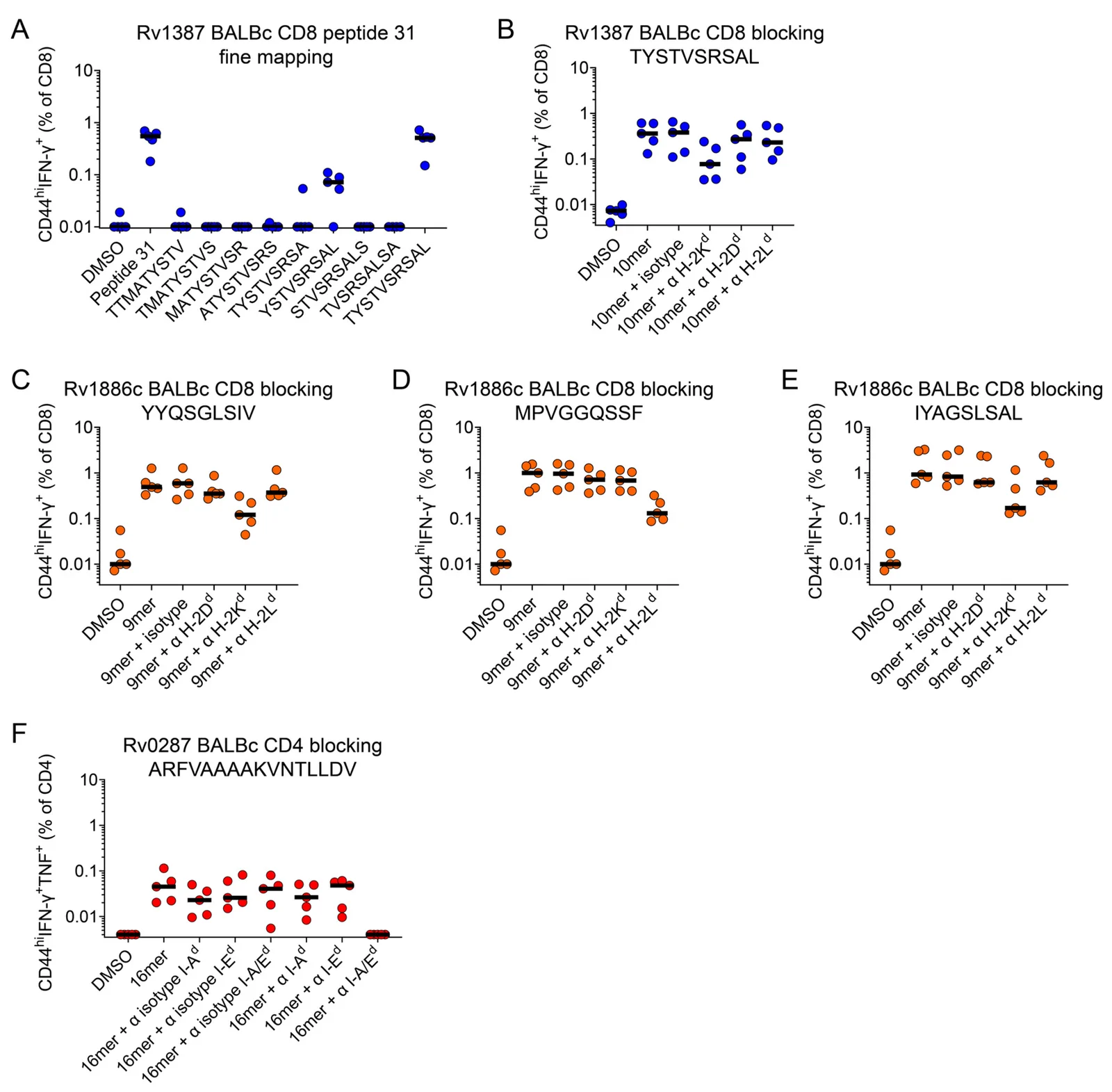
MHC restriction of immunodominant CD4 and CD8 T-cell epitopes in BALB/c mice. (**A**) CD8 responses in BALB/c splenocytes after 50 μg Rv1387 DNA prime-boost immunization and ex vivo stimulation with Rv1387 peptide pool, Rv1387 peptide 31, overlapping 9-mer peptides spanning peptide 31, or the predicted 10-mer peptide. (**B**) MHC class I restriction analysis of Rv1387 10-mer peptide using blocking antibodies against H-2K^d^, H-2D^d^, and H-2L^d^, and isotype antibody control. (**C**–**E**) MHC class I restriction analysis of immunogenic Rv1886c CD8 T-cell 9mers using blocking antibodies against H-2K^d^, H-2D^d^, and H-2L^d^, and isotype antibody control. (**F**) MHC class II restriction analysis of immunogenic Rv0287 peptide 12 using blocking antibodies against I-A^d^, I-E^d^, and I-A^d^/E^d^, and isotype antibody controls. For (**A**,**B**), groups of n = 5 mouse splenocytes stimulated with the indicated peptides were compared to DMSO negative control and overlapping peptide pool positive control. For (**C**–**F**), groups of n = 5 mouse splenocytes stimulated with the indicated peptides and incubated with the indicated blocking antibody were compared to isotype antibody control. Dots represent individual mice and bars indicate medians.

**Figure 3:**
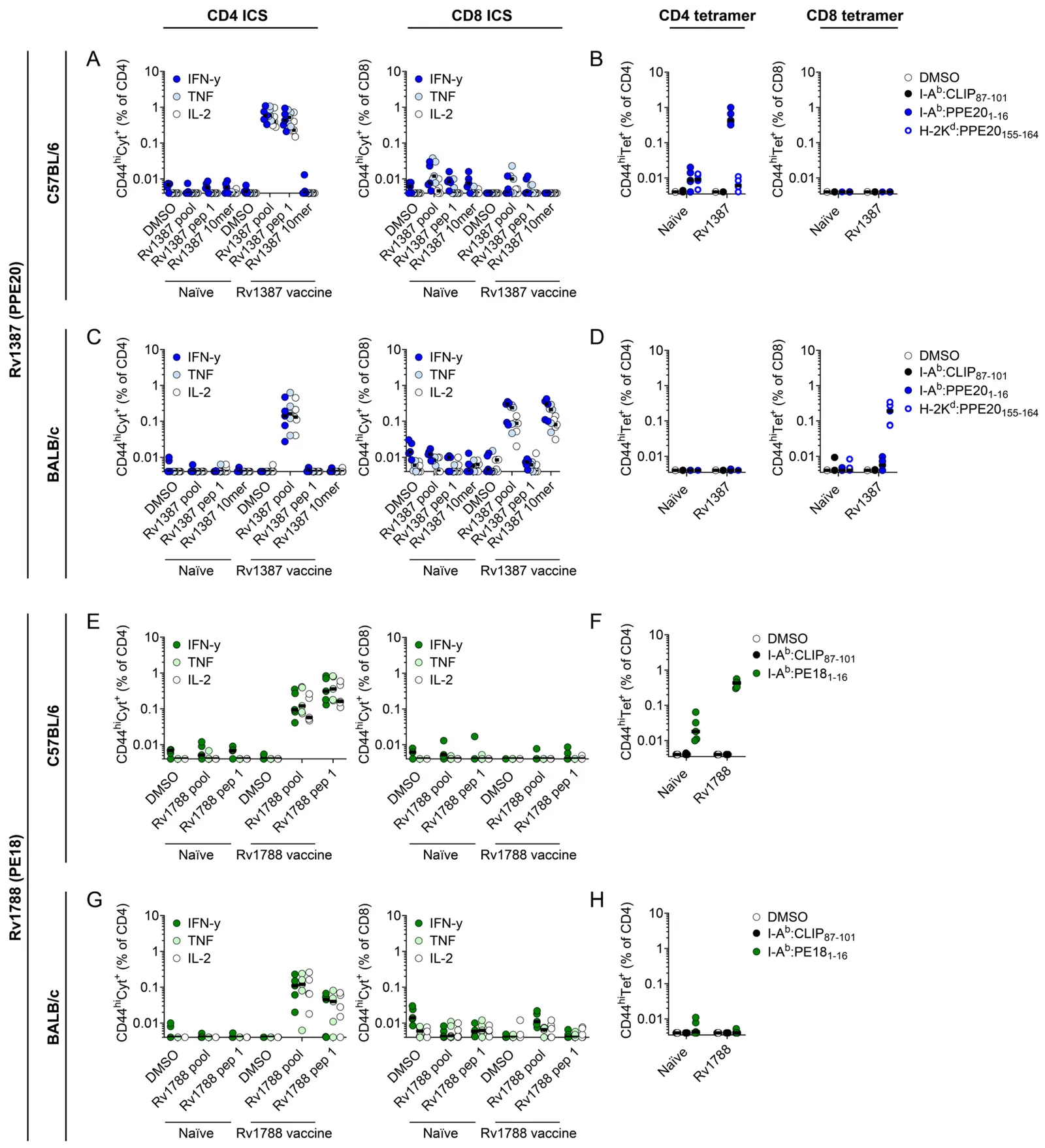
Validation of Rv1387 and Rv1788 tetramers in C57BL/6 and BALB/c mice. (**A**) CD4 and CD8 responses in naïve and vaccinated C57BL/6 splenocytes after 50 μg Rv1387 prime-boost DNA vaccination and ex vivo stimulation with the Rv1387 peptide pool, peptide 1, or the peptide 31 10-mer peptide. (**B**) Tetramer staining of the same mice from (**A**). (**C**) CD4 and CD8 responses in naïve and vaccinated BALB/c splenocytes after Rv1387 DNA vaccination and stimulation with the Rv1387 peptide pool, peptide 1, or the peptide 31 10-mer peptide. (**D**) Tetramer staining of the same mice from (**C**). (**E**) CD4 and CD8 responses in naïve and C57BL/6 splenocytes after Rv1788 DNA vaccination and stimulation with the Rv1788 peptide pool or peptide 1. (**F**) Tetramer staining of the same mice from (**E**). (**G**) CD4 and CD8 responses in naïve and BALB/c splenocytes after Rv1788 DNA prime-boost vaccination and stimulation with the Rv1788 peptide pool or peptide 1. (**H**) Tetramer staining of the same mice from (**G**). For all ICS studies (**A**,**C**,**E**,**G**), groups of n = 5 vaccinated mouse splenocytes stimulated with the indicated peptides were compared to the DMSO stimulated negative control and antigen overlapping peptide pool positive control. For all tetramer studies (**B**,**D**,**F**,**H**), groups of n = 5 vaccinated mouse splenocytes were compared naïve mouse splenocytes. Dots represent individual mice and bars indicate medians.

**Figure 4:**
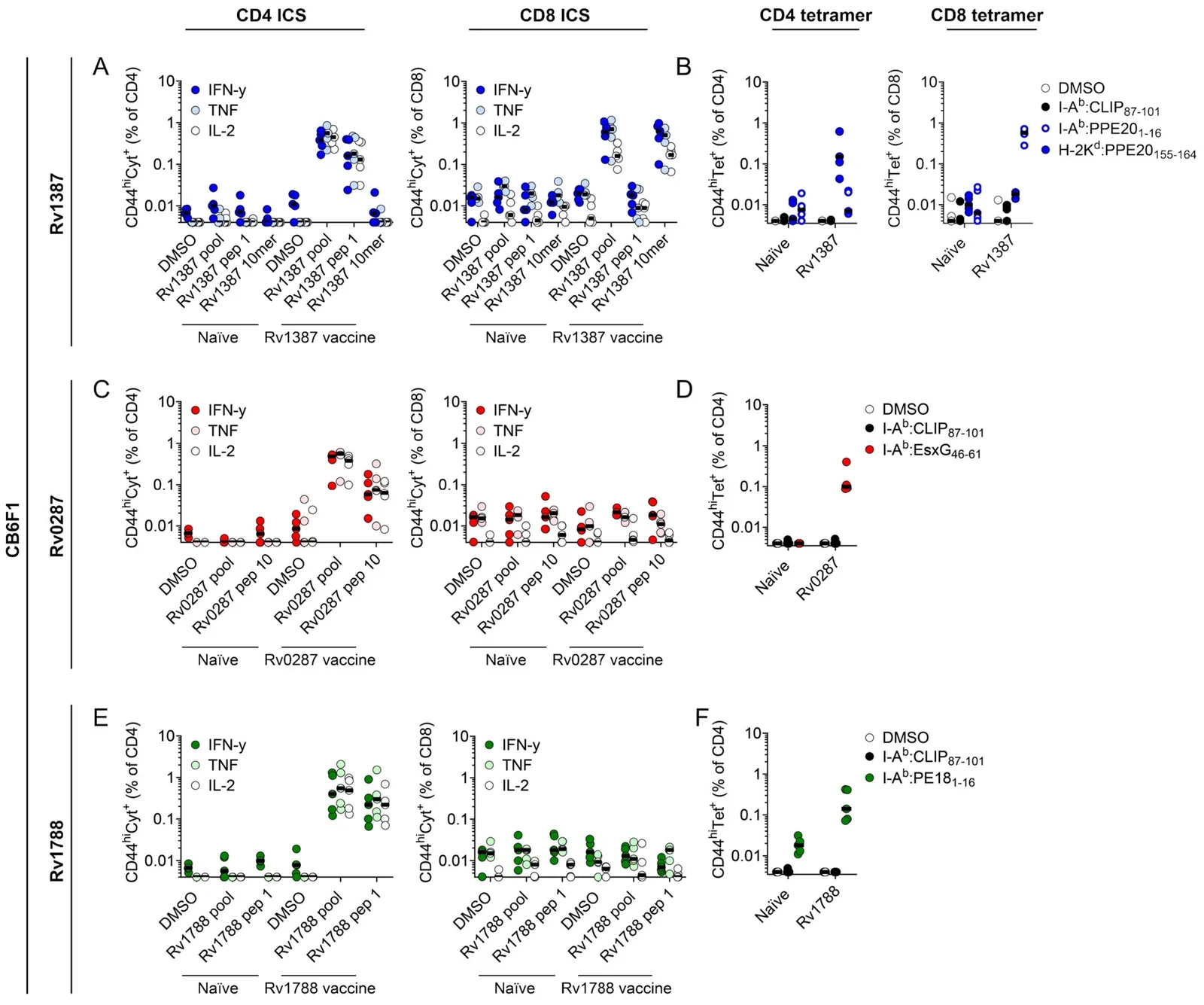
Validation of Rv1387, Rv0287, and Rv1788 tetramers in CB6F1 mice. (**A**) CD4 and CD8 responses in naïve and vaccinated CB6F1 splenocytes after 50 μg Rv1387 prime-boost DNA vaccination and ex vivo stimulation with the Rv1387 peptide pool, peptide 1, or the peptide 31 10-mer peptide. (**B**) Tetramer staining in the same mice from (**A**). (**C**) CD4 and CD8 responses in naïve and vaccinated CB6F1 splenocytes after Rv0287 DNA vaccination and stimulation with the Rv0287 peptide pool or peptide 10. (**D**) Tetramer staining in the same mice from (**C**). (**E**) CD4 and CD8 responses in naïve and CB6F1 splenocytes after Rv1788 DNA vaccination and stimulation with the Rv1788 peptide pool or peptide 1. (**F**) Tetramer staining in the same mice from (**E**). For all ICS studies (**A**,**C**,**E**), groups of n = 5 vaccinated mouse splenocytes stimulated with the indicated peptides were compared to the DMSO stimulated negative control and antigen overlapping peptide pool positive control. For all tetramer studies (**B**,**D**,**F**), groups of n = 5 vaccinated mouse splenocytes were compared naïve mouse splenocytes. Dots represent individual mice and bars indicate medians.

**Figure 5:**
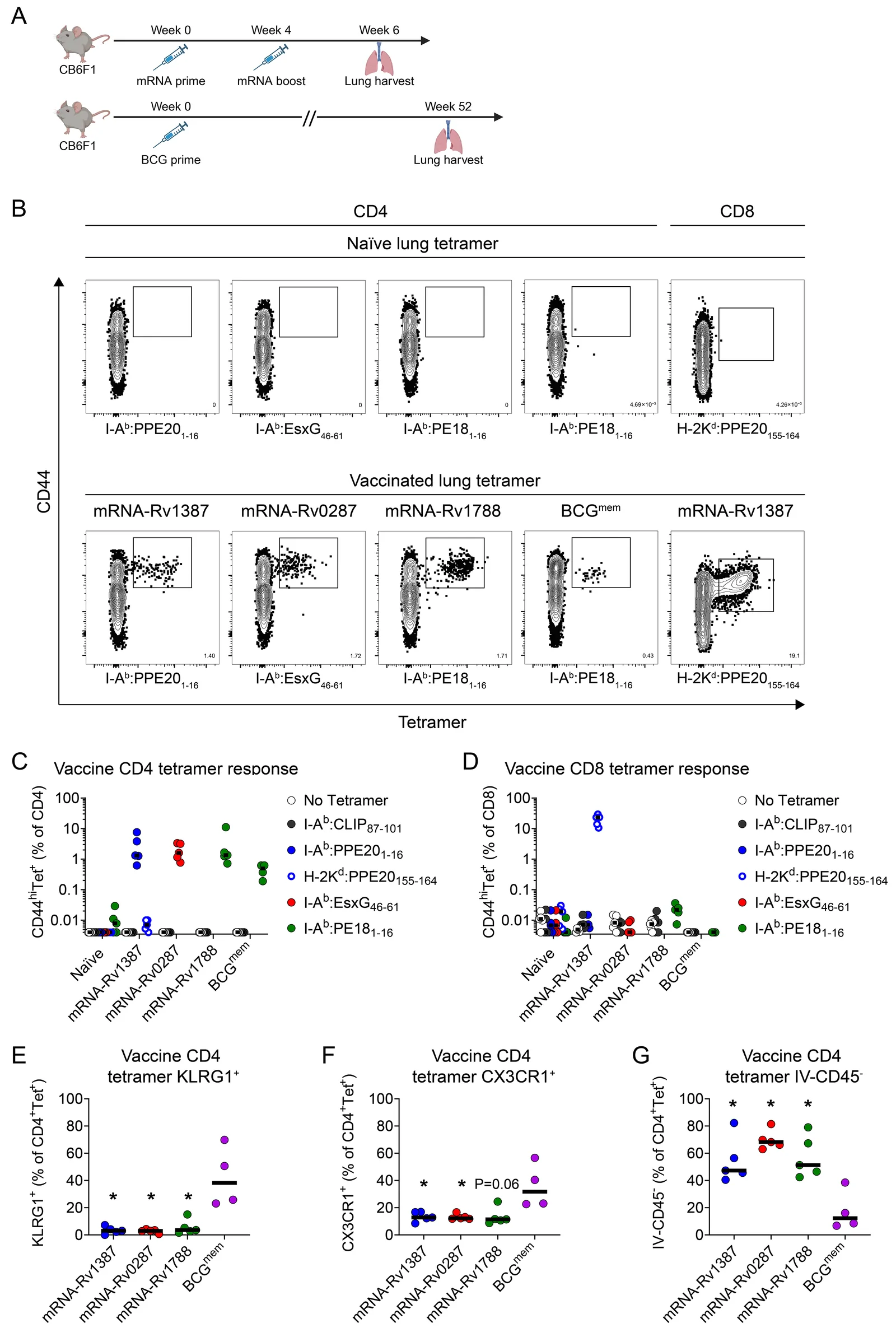
Lung mucosal immunology of antigen-specific T cells induced by mRNA-LNP and BCG^mem^ vaccination in CB6F1 mice. (**A**) Study scheme. (**B**) Representative lung tetramer staining flow cytometry plots from all tetramers and vaccine groups. (**C**,**D**) Tetramer staining of lung antigen-specific CD4 and CD8 T cells following 5 μg mRNA-LNP prime-boost or BCG^mem^ immunization in CB6F1 mice. KLRG1 (**E**), CX3CR1 (**F**), and IV-CD45 (**G**) staining frequencies among tetramer-positive antigen-specific CD4 T cells following mRNA-LNP or BCG^mem^ immunization. In (**C**,**D**), groups of n = 4–5 vaccinated mouse lung lymphocytes stained with tetramer were compared to n = 5 naïve mice. In (**E**–**G**), a group n = 4 BCG^mem^ vaccinated mouse lung lymphocytes was compared to groups of n = 5 mRNA vaccinated mice. Dots represent individual mice and bars indicate medians. * Represents *p* < 0.05 by Mann–Whitney U test relative to the BCG group.

## Data Availability

The original contributions presented in this study are included in the article/Supplementary Materials. Further inquiries can be directed to the corresponding author.

## Supplementary Materials

The following supporting information can be downloaded at: https://www.mdpi.com/article/10.3390/vaccines14100866/s1, Figure S1. Rv1387 and Rv1886c matrix subpool testing in C57BL/6 and BALB/c mice. (A) Rv1387 matrix subpools inducing CD4 T cell responses in C57BL/6 mice after 50 μg Rv1387 DNA prime-boost vaccination, ex vivo splenocyte stimulation, and flow cytometry with ICS. (B) Rv1387 matrix subpools inducing CD8 T cell responses in C57BL/6 mice after 50 μg Rv1387 DNA prime-boost vaccination, ex vivo splenocyte stimulation, and flow cytometry with ICS. (C) Rv1387 matrix subpools inducing CD4 T cell responses in BALBc mice after 50 μg Rv1387 DNA prime-boost vaccination, ex vivo splenocyte stimulation, and flow cytometry with ICS. (D) Rv1387 matrix subpools inducing CD8 T cell responses in BALBc mice after 50 μg Rv1387 DNA prime-boost vaccination, ex vivo splenocyte stimulation, and flow cytometry with ICS. (E) Rv1886c matrix subpools inducing CD4 T cell responses in C57BL/6 mice after 50 μg Rv1886c DNA prime-boost vaccination, ex vivo splenocyte stimulation, and flow cytometry with ICS. (F) Rv1886c matrix subpools inducing CD8 T cell responses in C57BL/6 mice after 50 μg Rv1886c DNA prime-boost vaccination, ex vivo splenocyte stimulation, and flow cytometry with ICS. (G) Rv1886c matrix subpools inducing CD4 T cell responses in BALBc mice after 50 μg Rv1886c DNA prime-boost vaccination, ex vivo splenocyte stimulation, and flow cytometry with ICS. (H) Rv1886c matrix subpools inducing CD8 T cell responses in BALBc mice after 50 μg Rv1886c DNA prime-boost vaccination, ex vivo splenocyte stimulation, and flow cytometry with ICS. For all experiments, groups of n = 4–5 mouse splenocytes stimulated with the indicated matrix subpools were compared to DMSO negative control and overlapping peptide pool positive control. Dots represent individual mice and red bars indicate medians. *p* values represent Mann–Whitney U tests for the indicated peptide compared to DMSO control. * Represents *p* < 0.05 and ** represents *p* < 0.01. Figure S2. Validation of the previously described Rv0287 tetramer in C57BL/6 and BALB/c mice. (A) CD4 and CD8 T cell responses in naïve and vaccinated C57BL/6 splenocytes after 50 μg Rv0287 prime-boost DNA vaccination and ex vivo stimulation with the Rv0287 peptide pool or peptide 10. (B) Tetramer staining of the same mice from (A). (C) CD4 and CD8 responses in naïve and vaccinated BALB/c splenocytes after Rv0287 DNA vaccination and stimulation with the Rv0287 peptide pool or peptide 10. (D) Tetramer staining of the same mice from (C). For all ICS studies (A,C), groups of n = 5 vaccinated mouse splenocytes stimulated with the indicated peptides were compared to the DMSO stimulated negative control and antigen overlapping peptide pool positive control. For all tetramer studies (B,D), groups of n = 5 vaccinated mouse splenocytes were compared naïve mouse splenocytes. Dots represent individual mice and bars indicate medians. Figure S3. Gating strategy for the identification of T cell populations. Table S1. Rv1387 16mer peptides, Rv0287 16mer peptides, Rv1886c 16mer peptides and linear subpools, and Rv1788 16mer peptides. Table S2. Rv1387 matrix subpools. Table S3. Rv1387 peptide 31 minimal CD8 T cell epitope and MHC class I restriction predictions. Table S4. Rv1886c CD8 T cell epitope MHC class I restriction predictions. Table S5. Rv0287 peptide 12 CD4 T cell epitope and MHC class II restriction predictions.
