# Supplementary material for: Epitope Mapping and MHC Restriction of Next-Generation Tuberculosis Vaccine Antigens in Mice
Source: Vaccines (Basel). 2026 Oct 1;14(10):866. doi: 10.3390/vaccines14100866 (PMC13709347; doi:10.3390/vaccines14100866)
Supplement: Supplementary file 1 [file vaccines-14-00866-s001.zip › vaccines-4506055-supplementary.pdf]

Figure S1

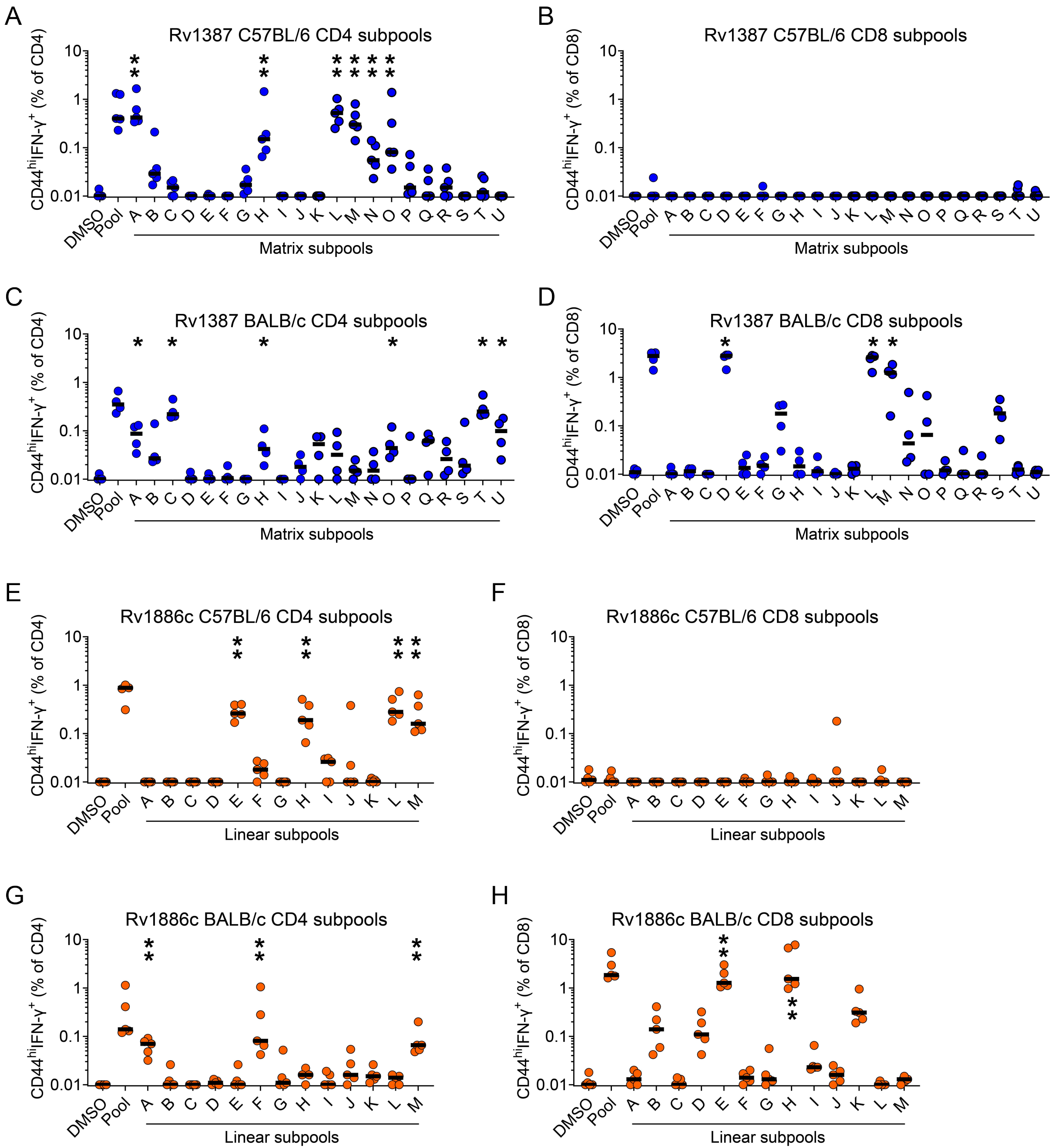
A
Rv1387 C57BL/6 CD4 subpools
CD44hiIFN-γ+ (% of CD4)
Matrix subpools
B
Rv1387 C57BL/6 CD8 subpools
CD44hiIFN-γ+ (% of CD8)
Matrix subpools
C
Rv1387 BALB/c CD4 subpools
CD44hiIFN-γ+ (% of CD4)
Matrix subpools
D
Rv1387 BALB/c CD8 subpools
CD44hiIFN-γ+ (% of CD8)
Matrix subpools
E
Rv1886c C57BL/6 CD4 subpools
CD44hiIFN-γ+ (% of CD4)
Linear subpools
F
Rv1886c C57BL/6 CD8 subpools
CD44hiIFN-γ+ (% of CD8)
Linear subpools
G
Rv1886c BALB/c CD4 subpools
CD44hiIFN-γ+ (% of CD4)
Linear subpools
H
Rv1886c BALB/c CD8 subpools
CD44hiIFN-γ+ (% of CD8)
Linear subpools
DMSO
Pool
10
1
0.1
0.01

Figure S2

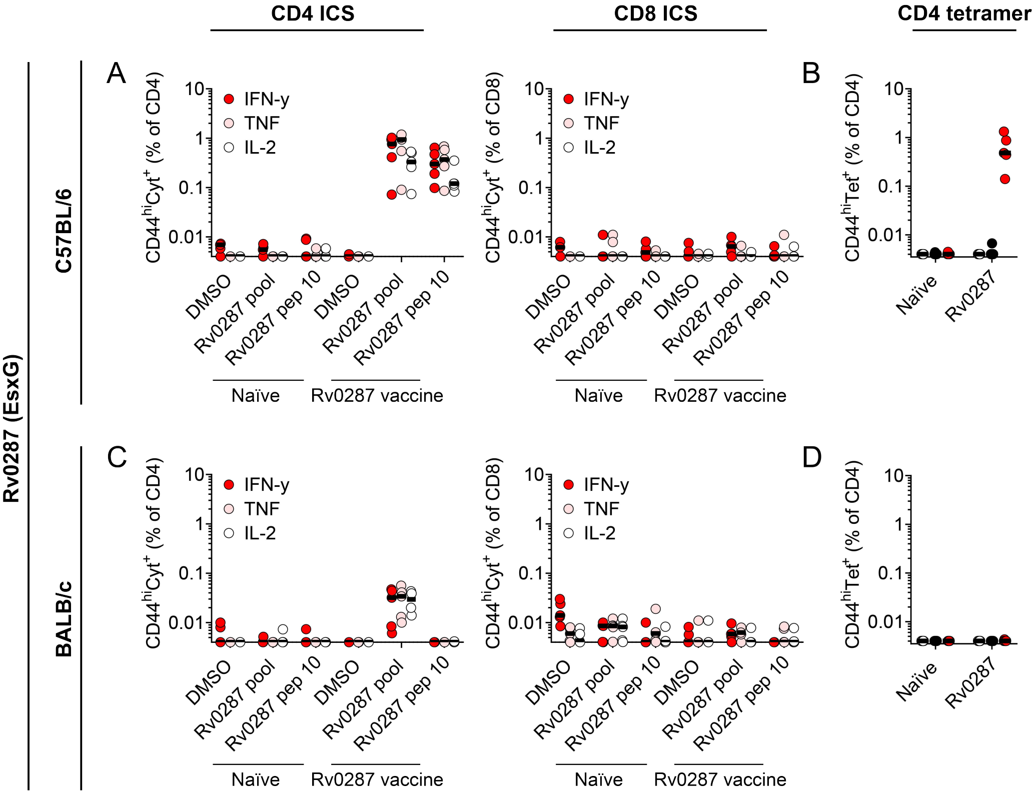

CD4 ICS
CD8 ICS
CD4 tetramer
Rv0287 (EsxG)
C57BL/6
BALB/c
A
B
C
D
CD44hiCyt+ (% of CD4)
CD44hiCyt+ (% of CD8)
CD44hiTet+ (% of CD4)
10
1
0.1
0.01
IFN-y
TNF
IL-2
DMSO
Rv0287 pool
Rv0287 pep 10
Naïve
Rv0287 vaccine
Rv0287

Figure S3

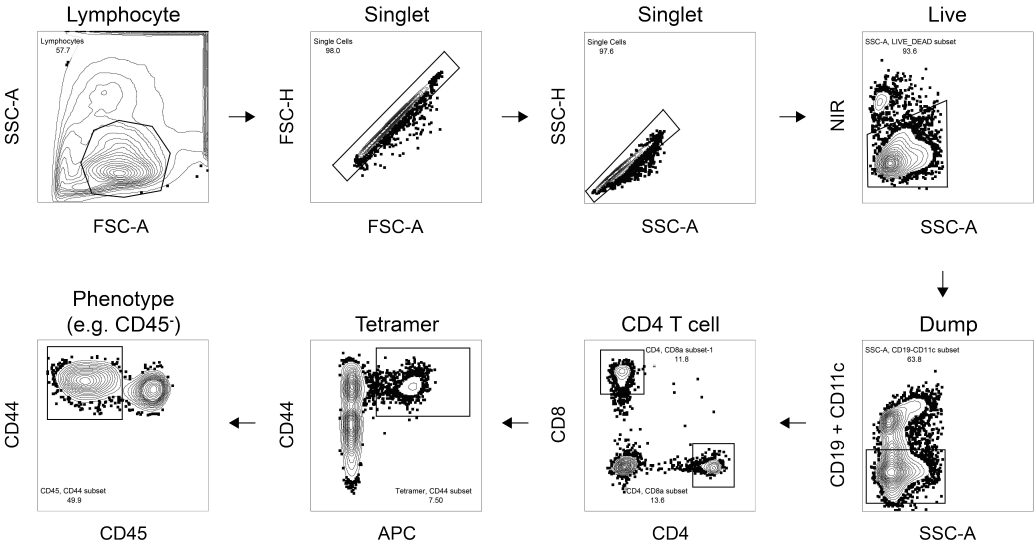
Lymphocyte
Lymphocytes
57.7
SSC-A
FSC-A
Singlet
Single Cells
98.0
FSC-H
FSC-A
Singlet
Single Cells
97.6
SSC-H
SSC-A
Live
SSC-A, LIVE_DEAD subset
93.6
NIR
SSC-A
Dump
SSC-A, CD19-CD11c subset
63.8
CD19 + CD11c
SSC-A
CD4 T cell
CD4, CD8a subset-1
11.8
CD4, CD8a subset
13.6
CD8
CD4
Tetramer
Tetramer, CD44 subset
7.50
CD44
APC
Phenotype
(e.g. CD45-)
CD45, CD44 subset
49.9
CD44
CD45

Table S1

Rv1387

Peptide number	Peptide sequence
1	MTEPWIAFPPEVHSAM
2	IAFPPEVHSAMLNYGA
3	EVHSAMLNYGAGVGPM
4	MLNYGAGVGPMLISAT
5	AGVGPMLISATQNGEL
6	MLISATQNGELSAQYA
7	TQNGELSAQYAEAASE
8	LSAQYAEAASEVEELL
9	AEAASEVEELLGVVAS
10	EVEELLGVVASEGWQG
11	LGVVASEGWQGQAAEA
12	SEGWQGQAAEAFVAAY
13	GQAAEAFVAAYMPFLA
14	AFVAAYMPFLAWLIQA
15	YMPFLAWLIQASADCV
16	AWLIQASADCVEMAAQ
17	ASADCVEMAAQQHVVI
18	VEMAAQQHVVIEAYTA
19	QQHVVIEAYTAAVELM
20	IEAYTAAVELMPTQVE
21	AAVELMPTQVELAANQ
22	MPTQVELAANQIKLAV
23	ELAANQIKLAVLVATN
24	QIKLAVLVATNFFGIN
25	VLVATNFFGINTIPIA
26	NFFGINTIPIAINEAE
27	NTIPIAINEAEYVEMW
28	AINEAEYVEMWVRAAT
29	EYVEMWVRAATTMATY
30	WVRAATTMATYSTVSR
31	TTMATYSTVSRSALSA
32	YSTVSRSALSAMPHTS
33	RSALSAMPHTSPPPLI
34	AMPHTSPPPLILKSDE
35	SPPPLILKSDELLPDT
36	ILKSDELLPDTGEDSD
37	ELLPDTGEDSDEDGHN
38	TGEDSDEDGHNHGGHS
39	DEDGHNHGGHSHGGHA
40	NHGGHSHGGHARMIDN
41	SHGGHARMIDNFFAEI
42	ARMIDNFFAEILRGVS
43	NFFAEILRGVSAGRIV
44	ILRGVSAGRIVWDPVN
45	SAGRIVWDPVNGTLNG
46	VWDPVNGTLNGLDYDD
47	NGTLNGLDYDDYVYPG
48	GLDYDDYVYPGHAIWW
49	DYVYPGHAIWWLARGL
50	GHAIWWLARGLEFFQD
51	WLARGLEFFQDGEQFG
52	LEFFQDGEQFGELLFT
53	DGEQFGELLFTNPTGA
54	GELLFTNPTGAFQFLL
55	TNPTGAFQFLLYVVVV
56	AFQFLLYVVVVDLPTH
57	LYVVVVDLPTHIAQIA
58	VDLPTHIAQIATWLGQ
59	HIAQIATWLGQYPQLL
60	ATWLGQYPQLLSAALT
61	QYPQLLSAALTGVIAH
62	LSAALTGVIAHLGAIT
63	TGVIAHLGAITGLAGL

Rv0287

Peptide number	Peptide sequence
1	MSLLDAHIPQLVASQS
2	AHIPQLVASQSAFAAK
3	LVASQSAFAAKAGLMR
4	SAFAAKAGLMRHTIGQ
5	KAGLMRHTIGQAEQAA
6	RHTIGQAEQAAMSAQA
7	QAEQAAMSAQAFHQGE
8	AMSAQAFHQGESSAAF
9	AFHQGESSAAFQAAHA
10	ESSAAFQAAHARFVAA
11	FQAAHARFVAAAAKVN
12	ARFVAAAAKVNTLLDV
13	AAAKVNTLLDVAQANL
14	NTLLDVAQANLGEAAG
15	VAQANLGEAAGTYVAA
16	LGEAAGTYVAADAAAA
17	GTYVAADAAAASTYTGF

Rv1886c

Peptide number	Peptide sequence	Subpool
1	MTDVSRKIRAWGRRLM	A
2	RKIRAWGRRLMIGTAA	A
3	WGRRLMIGTAAAVVLP	A
4	MIGTAAAVVLPGLVGL	A
5	AAVVLPGLVGLAGGAA	A
6	PGLVGLAGGAATAGAF	B
7	LAGGAATAGAFSRPGL	B
8	ATAGAFSRPGLPVEYL	B
9	FSRPGLPVEYLQVPSP	B
10	LPVEYLQVPSPSMGRD	B
11	LQVPSPSMGRDIKVQF	C
12	PSMGRDIKVQFQSGGN	C
13	DIKVQFQSGGNNSPAV	C
14	FQSGGNNSPAVYLLDG	C
15	NNSPAVYLLDGLRAQD	C
16	VYLLDGLRAQDDYNGW	D
17	GLRAQDDYNGWDINTP	D
18	DDYNGWDINTPAFEWY	D
19	WDINTPAFEWYYQSGL	D
20	PAFEWYYQSGLSIVMP	D
21	YYQSGLSIVMPVGGQS	E
22	LSIVMPVGGQSSFYSD	E
23	PVGGQSSFYSDWYSPA	E
24	SSFYSDWYSPACGKAG	E
25	DWYSPACGKAGCQTYK	E
26	ACGKAGCQTYKWETFL	F
27	GCQTYKWETFLTSELP	F
28	KWETFLTSELPQWLSA	F
29	LTSELPQWLSANRAVK	F
30	PQWLSANRAVKPTGSA	F
31	ANRAVKPTGSAAIGLS	G
32	KPTGSAAIGLSMAGSS	G
33	AAIGLSMAGSSAMILA	G
34	SMAGSSAMILAAYHPQ	G
35	SAMILAAYHPQQFIYA	G
36	AAYHPQQFIYAGSLSA	H
37	QQFIYAGSLSALLDPS	H
38	AGSLSALLDPSQGMGP	H
39	ALLDPSQGMGPSLIGL	H
40	SQGMGPSLIGLAMGDA	H
41	PSLIGLAMGDAGGYKA	
42	LAMGDAGGYKAADMWG	

Rv1788

Peptide number	Peptide sequence
1	MSFVTTQPEALAAAAG
2	TQPEALAAAAGSLQGI
3	LAAAAGSLQGIGSALN
4	GSLQGIGSALNAQNAA
5	IGSALNAQNAAAATPT
6	NAQNAAAATPTTGVVP
7	AAATPTTGVVPAAADE
8	TTGVVPAAADEVSALT
9	PAAADEVSALTAAQFA
10	EVSALTAAQFAAHAQI
11	TAAQFAAHAQIYQAVS
12	AAHAQIYQAVSAQAAA
13	IYQAVSAQAAAIHEMF
14	SAQAAAIHEMFVNTLQ
15	AIHEMFVNTLQMSSGS
16	FVNTLQMSSGSYAATE
17	QMSSGSYAATEAANAA
18	SYAATEAANAAAAG

64	HLGAITGLAGLSGLSA
65	TGLAGLSGLSAIPSAA
66	LSGLSAIPSAAIPAVV
67	AIPSAAIPAVVPELTP
68	AIPAVVPELTPVAAAP
69	VPELTPVAAAPPMLAV
70	PVAAAPPMLAVAGVGP
71	PPMLAVAGVGPAVAAP
72	VAGVGPAVAAPGMLPA
73	PAVAAPGMLPASAPAP
74	PGMLPASAPAPAAAAG
75	ASAPAPAAAAGATAAG
76	PAAAAGATAAGPTPPA
77	GATAAGPTPPATGFGG
78	GPTPPATGFGGFPPYL
79	ATGFGGFPPYLVGGGG
80	GFPPYLVGGGGPGIGF
81	LVGGGGPGIGFGSGQS
82	GPGIGFGSGQSAHAKA
83	FGSGQSAHAKAAASDS
84	SAHAKAAASDSAAAES
85	AAASDSAAAESAAQAS
86	SAAAESAAQASARAQA
87	SAAQASARAQARAARR
88	SARAQARAARRGRSAA
89	ARAARRGRSAAKARGH
90	RGRSAAKARGHRDEFV
91	AKARGHRDEFVTMDMG
92	HRDEFVTMDMGFDAAA
93	VTMDMGFDAAAPAPEH
94	GFDAAAPAPEHQPGAR
95	APAPEHQPGARASDCG
96	HQPGARASDCGAGPIG
97	RASDCGAGPIGFAGTV
98	GAGPIGFAGTVRKEAV
99	GFAGTVRKEAVVKAAG
100	VRKEAVVKAAGLTTLA
101	VVKAAGLTTLAGDDFG
102	GLTTLAGDDFGGGPTM
103	AGDDFGGGPTMPMMPG
104	GGGPTMPMMPGTWTHD
105	MPMMPGTWTHDQGVFD
106	GTWTHDQGVFD

43	AGGYKAADMWGPSSDP	I
44	AADMWGPSSDPAWERN	
45	GPSSDPAWERNDPTQQ	
46	PAWERNDPTQQIPKLV	
47	NDPTQQIPKLVANNTR	
48	QIPKLVANNTRLWVYC	J
49	VANNTRLWVYCGNGTP	
50	RLWVYCGNGTPNELGG	
51	CGNGTPNELGGANIPA	
52	PNELGGANIPAEFLEN	
53	GANIPAEFLENFVRSS	K
54	AEFLENFVRSSNLKFQ	
55	NFVRSSNLKFQDAYNA	
56	SNLKFQDAYNAAGGHN	
57	QDAYNAAGGHNAVFNF	
58	AAGGHNAVFNFPPNGT	L
59	NAVFNFPPNGTHSWEY	
60	FPPNGTHSWEYWGAQL	
61	THSWEYWGAQLNAMKG	
62	YWGAQLNAMKGDLQSS	M
63	LNAMKGDLQSSLGAG	

Table S2

	Rv1387 matrix subpools									
**Subpool**	L	M	N	O	P	Q	R	S	T	U
A	1	2	3	4	5	6	7	8	9	10
B	11	12	13	14	15	16	17	18	19	20
C	21	22	23	24	25	26	27	28	29	30
D	31	32	33	34	35	36	37	38	39	40
E	41	42	43	44	45	46	47	48	49	50
F	51	52	53	54	55	56	57	58	59	60
G	61	62	63	64	65	66	67	68	69	70
H	71	72	73	74	75	76	77	78	79	80
I	81	82	83	84	85	86	87	88	89	90
J	91	92	93	94	95	96	97	98	99	100
K	101	102	103	104	105	106				

Table S3

Rv1387 peptide 31 length predictions

Peptide length	Peptide	TAP score	MHC score	Processing score	Total score	MHC IC_{50}
10	TYSTVSRSAL	0.59	-2.26	2.02	-0.24	182.4
11	ATYSTVSRSAL	0.57	-2.92	2	-0.92	826.6
12	MATYSTVSRSAL	0.45	-3.1	1.88	-1.22	1250.5
13	TMATYSTVSRSAL	0.47	-3.17	1.9	-1.27	1483
14	TTMATYSTVSRSAL	0.44	-3.18	1.87	-1.31	1504.1
9	TTMATYSTV	0.16	-2.61	1.13	-1.49	411

Rv1387 peptide 31 binding allele predictions

Peptide	Peptide length	Allele	Median binding percentile	Netmhcpan_el core
TYSTVSRSAL	10	H2-Kd	0.12	TYSTVSSAL
TYSTVSRSA	9	H2-Kd	0.63	TYSTVSRSA
YSTVSRSAL	9	H2-Dd	0.78	YSTVSRSAL
YSTVSRSAL	9	H2-Kd	1.5	YSTVSRSAL
STVSRSAL	8	H2-Dd	1.6	STVSR-SAL

Table S4

Rv1886c peptide 21 binding allele predictions

Peptide	Peptide length	Allele	Median binding percentile	Netmhcpan_el core
YYQSGLSIV	9	H2-Kd	0.01	YYQSGLSIV
YYQSGLSI	8	H2-Kd	0.03	YYQ-SGLSI
YQSGLSIV	8	H2-Kd	2	-YQSGLSIV
YYQSGLSIV	9	H2-Dd	5.8	YYQSGLSIV
YYQSGLSI	8	H2-Dd	5.9	YYQ-SGLSI

Rv1886c peptide 22 binding allele predictions

Peptide	Peptide length	Allele	Median binding percentile	Netmhcpan_el core
MPVGGQSSF	9	H2-Ld	0.05	MPVGGQSSF
MPVGGQSSF	9	H2-Kd	2.3	MPVGGQSSF
PVGGQSSF	8	H2-Ld	4.7	-PVGGQSSF
MPVGGQSSF	9	H2-Dd	5.7	MPVGGQSSF
PVGGQSSF	8	H2-Dd	28	PVG-GQSSF

Rv1886c peptide 37 binding allele predictions

Peptide	Peptide length	Allele	Median binding percentile	Netmhcpan_el core
IYAGSLSAL	9	H2-Kd	0.01	IYAGSLSAL
YAGSLSAL	8	H2-Kd	0.74	-YAGSLSAL
YAGSLSAL	8	H2-Ld	3.2	YAGSL-SAL
IYAGSLSAL	9	H2-Dd	3.2	IYAGSLSAL
YAGSLSAL	8	H2-Dd	3.6	YAGS-LSAL

Table S5

Rv0287 peptide 12 binding allele predictions				
Peptide	Peptide length	Allele	Median binding percentile	Netmhcpan_el core
ARFVAAAKVNTL	13	H2-IEd	7.1	FVAAAKVN
ARFVAAAKVNTL	13	H2-IAd	7.8	FVAAAKVN
ARFVAAAKVNTLLD	15	H2-IAd	8	AAAAKVNTL
ARFVAAAKVNTLLDV	16	H2-IAd	9.9	AAAAKVNTL
ARFVAAAKVNTLL	14	H2-IAd	11	AAAAKVNTL
ARFVAAAKVNTLL	14	H2-IEd	17	FVAAAKVN
ARFVAAAKVNTLLD	15	H2-IEd	22	FVAAAKVN
ARFVAAAKVNTLLDV	16	H2-IEd	37	FVAAAKVN
